# DNA-Binding Proteins Essential for Protein-Primed Bacteriophage Φ29 DNA Replication

**DOI:** 10.3389/fmolb.2016.00037

**Published:** 2016-08-05

**Authors:** Margarita Salas, Isabel Holguera, Modesto Redrejo-Rodríguez, Miguel de Vega

**Affiliations:** Centro de Biología Molecular Severo Ochoa (Consejo Superior de Investigaciones Científicas), Universidad Autónoma de MadridMadrid, Spain

**Keywords:** bacteriophage Φ29, DNA replication, DNA polymerase, terminal protein, DNA binding proteins

## Abstract

*Bacillus subtilis* phage Φ29 has a linear, double-stranded DNA 19 kb long with an inverted terminal repeat of 6 nucleotides and a protein covalently linked to the 5′ ends of the DNA. This protein, called terminal protein (TP), is the primer for the initiation of replication, a reaction catalyzed by the viral DNA polymerase at the two DNA ends. The DNA polymerase further elongates the nascent DNA chain in a processive manner, coupling strand displacement with elongation. The viral protein p5 is a single-stranded DNA binding protein (SSB) that binds to the single strands generated by strand displacement during the elongation process. Viral protein p6 is a double-stranded DNA binding protein (DBP) that preferentially binds to the origins of replication at the Φ29 DNA ends and is required for the initiation of replication. Both SSB and DBP are essential for Φ29 DNA amplification. This review focuses on the role of these phage DNA-binding proteins in Φ29 DNA replication both *in vitro* and *in vivo*, as well as on the implication of several *B. subtilis* DNA-binding proteins in different processes of the viral cycle. We will revise the enzymatic activities of the Φ29 DNA polymerase: TP-deoxynucleotidylation, processive DNA polymerization coupled to strand displacement, 3′–5′ exonucleolysis and pyrophosphorolysis. The resolution of the Φ29 DNA polymerase structure has shed light on the translocation mechanism and the determinants responsible for processivity and strand displacement. These two properties have made Φ29 DNA polymerase one of the main enzymes used in the current DNA amplification technologies. The determination of the structure of Φ29 TP revealed the existence of three domains: the priming domain, where the primer residue Ser232, as well as Phe230, involved in the determination of the initiating nucleotide, are located, the intermediate domain, involved in DNA polymerase binding, and the N-terminal domain, responsible for DNA binding and localization of the TP at the bacterial nucleoid, where viral DNA replication takes place. The biochemical properties of the Φ29 DBP and SSB and their function in the initiation and elongation of Φ29 DNA replication, respectively, will be described.

## Introduction

Bacteriophages are the most abundant biological entities on earth (Brüssow and Hendrix, 2002). Approximately 96% of the reported bacteriophages belong to the order *Caudovirales*, which is composed of three families: *Myoviridae, Siphoviridae*, and *Podoviridae* (Ackermann, 2003). *Bacillus subtilis* phage Φ29 belongs to the *Podoviridae* family and to the Φ*29-like* genus, together with phages Φ15, PZA, BS32, B103, Nf, M2Y, and GA-1 (Ackermann, 1998). These are the smallest phages that infect *Bacillus*, and they are among the smallest known phages that possess a dsDNA genome (Anderson and Reilly, 1993). Based on its relatedness, these phages have been classified in three groups: group I includes phages Φ29, PZA, Φ15, and BS32; group II contains phages B103, Nf and M2Y; and group III has GA-1 as its only member (Yoshikawa et al., 1985, 1986; Pecenkova and Paces, 1999).

Bacteriophage Φ29 genome consists of a linear dsDNA ~19 Kb-long with a terminal protein (TP) covalently linked to each 5′ end (Salas, 1991). Φ29 has served as a model system for studying the protein-priming mechanism of DNA replication, being the TP-primed replication system best characterized *in vitro*. The use of a TP as primer for viral DNA replication has also been described for other bacteriophages (e.g., *Escherichia coli* and *Streptococcus pneumoniae* phages PRD1 and Cp-1, respectively), eukaryotic viruses (adenovirus), and some *Streptomyces* spp. (Chang and Cohen, 1994; Bao and Cohen, 2001). In addition, the presence of TPs has been described or suggested in viruses infecting *Archaea* (Bath et al., 2006; Peng et al., 2007), some linear plasmids of bacteria, fungi, and higher plants (Salas, 1991; Meinhardt et al., 1997; Chaconas and Chen, 2005), transposable elements (Kapitonov and Jurka, 2006) and mitochondrial DNA (Fricova et al., 2010).

Besides the essential role of priming DNA replication, TPs can perform additional functions. It has been shown that adenovirus TP is important for the anchoring of the viral genome to the nuclear matrix, which enhances transcription of the viral DNA (Schaack et al., 1990). TPs have also been shown to be required for DNA packaging (Bjornsti et al., 1982, 1983), transfection (Hirokawa, 1972; Ronda et al., 1983; Porter and Dyall-Smith, 2008), and nucleoid and nuclear targeting (Tsai et al., 2008; Muñoz-Espín et al., 2010; Redrejo-Rodríguez et al., 2012). Furthermore, biochemical studies have suggested that Φ29 TP is endowed with peptidoglycan-hydrolytic activity (Moak and Molineux, 2004).

## Φ29 terminal protein

**Figure 1:**
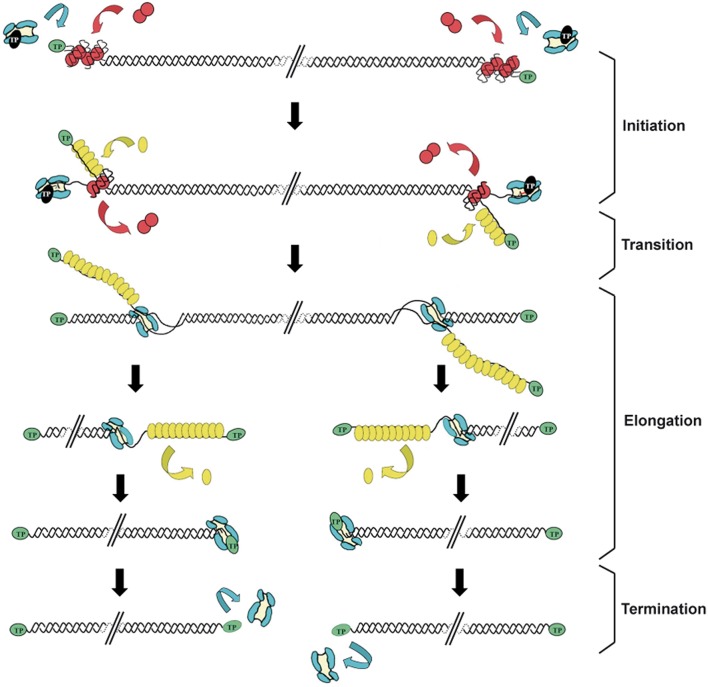
**Schematic representation of the bacteriophage Φ29 DNA replication mechanism**. Φ29 DNA replication starts non-simultaneously at both DNA ends. The TP/DNA polymerase heterodimer recognizes the p6-complexed replication origins and the DNA polymerase catalyzes the covalent linkage of dAMP to TP residue Ser232 (initiation reaction). After a transition step (not drawn in the figure), the DNA polymerase dissociates from the TP and continues processive elongation coupled to strand displacement. Viral protein p5 binds to the displaced ssDNA strands and is further removed during the polymerization process. Continuous elongation by two DNA polymerases gives rise to the complete duplication of the parental strands. Green ovals: parental TP; black ovals: primer TP; red circles: p6; blue: DNA polymerase; yellow ovals: SSB p5. Linear dsDNA is shown as a double helix. Adapted from de Vega and Salas (2011).

Replication of the Φ29 genome takes place by a process of symmetrical replication in which both origins are used for initiation in a non-simultaneous manner (Blanco et al., 1989; Figure 1). The protein that acts as primer for the initiation of Φ29 DNA replication, the so-called TP, is a 266 amino acids protein encoded by the early viral gene *3*. The first step of Φ29 DNA replication is the formation of a heterodimer between a free molecule of TP (primer TP) and the DNA polymerase (Φ29 DNAP) (Blanco et al., 1987). Then, this complex recognizes the replication origins, located at both ends of the viral genome, by specific interactions with both the TP that is linked to the genome ends by a previous round of replication (parental TP) and DNA sequences (García et al., 1984; Gutiérrez et al., 1986a,b; González-Huici et al., 2000a,b). The parental TP is the major signal for replication origin recognition by the heterodimer (Gutiérrez et al., 1986b; González-Huici et al., 2000b) and both, DNA polymerase and primer TP, are involved in such recognition through specific interactions with the parental TP (Freire et al., 1996; Illana et al., 1998; González-Huici et al., 2000a; Serna-Rico et al., 2000; Pérez-Arnáiz et al., 2007). The Φ29 double-stranded DNA binding protein p6 (DBP) (see below) binds all along Φ29 DNA forming a nucleoprotein complex that causes the unwinding of the DNA helix at the ends, facilitating the initiation step (Serrano et al., 1994). After origin recognition, the viral DNA polymerase catalyzes the formation of a phosphoester between the first dAMP and the hydroxyl group of the primer TP residue Ser232 (Blanco and Salas, 1984; Hermoso et al., 1985). The initiation reaction is directed by the second T at the 3′ end of the template (3′ TTTCAT 5′), after which the TP-dAMP complex translocates one position backwards to recover the information corresponding to the first T of the template strand. Then, the second T will serve again as template for the incorporation of the following nucleotide (Méndez et al., 1992). This backward translocation of the TP-dAMP complex is known as sliding-back mechanism and requires a terminal repetition of at least 2 nucleotides in the template strand to guarantee the fidelity of the initiation reaction (Méndez et al., 1992) (see below). The TP/DNA polymerase heterodimer is not dissociated immediately after initiation. There is a transition stage in which the DNA polymerase synthesizes a 5 nt-long elongation product while complexed with TP, undergoes some structural changes during the incorporation of nucleotides 6 to 9, and finally dissociates from the TP after the incorporation of the 10th nucleotide (Méndez et al., 1997). Then, the viral DNA polymerase continues DNA elongation in a processive manner, which occurs coupled to the displacement of the non-template strand (Blanco et al., 1989). DNA elongation by one Φ29 DNAP coming from each origin generates type I replicative intermediates, consisting of full-length Φ29 dsDNA molecules with two branches of ssDNA. These stretches of ssDNA are bound by the viral single-stranded DNA-binding protein p5 (SSB) (see below) (Gutiérrez et al., 1991), which will be further removed during the polymerization process. When the two replication forks meet, the type I replicative intermediate gives rise to two physically separated type II replicative intermediates. These molecules consist of full-length Φ29 DNA in which a portion of the DNA starting from one end is dsDNA and the portion spanning to the other end is ssDNA (Harding and Ito, 1980; Inciarte et al., 1980). Termination of viral DNA replication occurs when the DNA polymerase reaches the template end, and after replication of the last nucleotide, dissociates from the viral genome.

**Figure 2:**
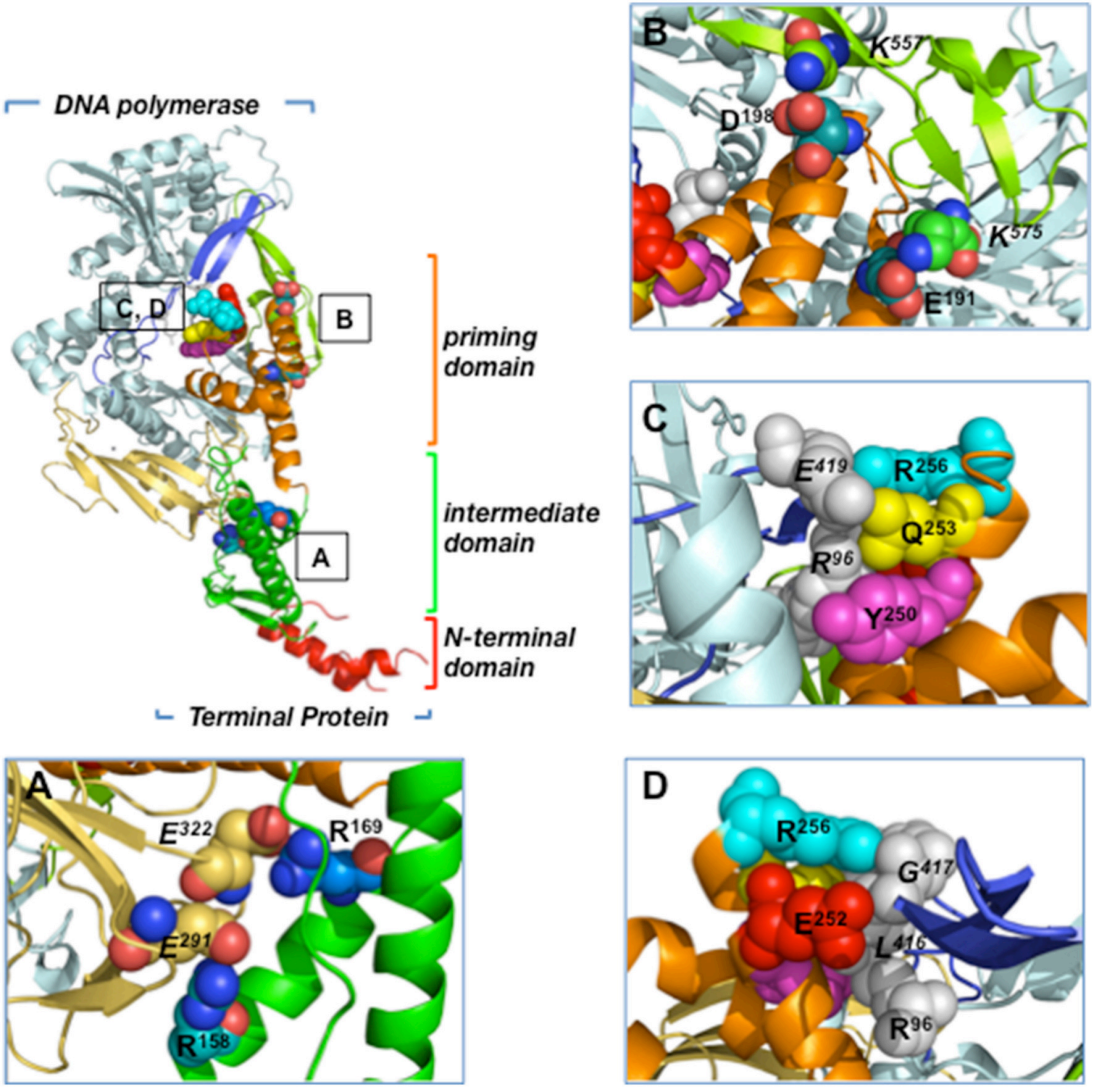
**Residues involved in Φ29 TP/DNA polymerase interaction**. Ribbon representation of the Φ29 TP/DNA polymerase heterodimer. DNA polymerase is colored in blue except for TPR1 (pale yellow), TPR2 (dark blue) and thumb (light green) subdomains. TP N-terminal, intermediate and priming domains are colored in red, green and orange, respectively. **(A)** Close-up view of the TP intermediate domain residues R158 and R169, proposed to make salt bridges with DNA polymerase residues E291 and E322 of the TPR1 subdomain, respectively. **(B)** Close-up view of the TP priming domain residues E191 and D198, proposed to interact with the DNA polymerase thumb subdomain residues K575 and K557, respectively. **(C)** The proposed stacking interactions between TP priming domain residues R256, Q253, and Y250, and DNA polymerase residues R96 (exonuclease domain) and E419 (TPR2 subdomain) are indicated. **(D)** Close-up view of the proposed interaction between TP priming domain residues R256 and E252 with DNA polymerase residues R96 (exonuclease domain) and L416 and G417 (TPR2 subdomain). Coordinates of the Φ29 TP/DNA polymerase heterodimer are from PDB ID 2EX3 (Kamtekar et al., 2006). The figure was made using the Pymol software (http://www.pymol.org).

The 3.0 Å resolution crystallographic structure of the heterodimer formed between Φ29 DNA polymerase and TP revealed that the latter is composed of three structural domains (Kamtekar et al., 2006; see Figure 2):

- The TP N-terminal domain comprises residues 1 to 73 and its tertiary structure is unknown because it was disordered in the crystal lattice. Circular dichroism experiments have shown that this domain has a high content in αhelix (60%), and secondary structure predictions determined two αhelices connected by a disordered loop (Holguera et al., 2014). This domain is responsible for non-sequence specific DNA binding (Zaballos and Salas, 1989) and for the localization of the protein at the bacterial nucleoid (Muñoz-Espín et al., 2010). In addition, a role in origin unwinding has been proposed for the TP N-terminal domain, since this domain is not required for the initiation reaction at a partially open origin (Pérez-Arnáiz et al., 2007; Gella et al., 2014).
- The TP intermediate domain (residues 74–172) is composed of two long αhelices and a short β-turn-β structure. This domain makes extensive contacts with the DNA polymerase (mainly with the TPR1 subdomain), being the main responsible for the specificity of the interaction with the DNA polymerase and for the stability of the heterodimer (Pérez-Arnáiz et al., 2007; del Prado et al., 2012).
- The TP C-terminal domain or priming domain (residues 173–266) is connected to the intermediate domain through a hinge region and it is comprised of a four-helix bundle. The TP priming residue Ser232 lies in the so-called priming loop, a disordered loop comprising residues 227–233. The TP priming domain makes extensive interactions with the TPR2 and thumb subdomains of the DNA polymerase (Kamtekar et al., 2006; del Prado et al., 2012) and is responsible for dictating the nucleotide used as template during initiation of viral DNA replication (Longás et al., 2008). The TP priming domain has been proposed to mimic duplex product DNA in its electrostatic profile and binding site in the DNA polymerase, as both occupy the same binding cleft in the DNA polymerase (de Vega et al., 1998a; Kamtekar et al., 2006).

There are not proteins in structural databases with sufficient structural homology with Φ29 TP. Genes encoding TPs from other Φ*29-like* phages such as B103, PZA, Nf, and GA-1 have been sequenced (Paces et al., 1985; Leavitt and Ito, 1987; Illana et al., 1996; Pecenková et al., 1997; Meijer et al., 2001). The amino acid sequence comparison of these TPs has revealed a high degree of conservation between PZA and Φ29 TPs (97.7% identity). In fact, Φ29 TP can functionally substitute for PZA TP *in vivo* (Bravo et al., 1994a). The conservation is lower in the case of Nf (62.4% identity) and B103 (62% identity) TPs (Leavitt and Ito, 1987; Pecenková et al., 1997). GA-1 TP is the most distantly related one, being the percentage of identity with Φ29 TP of 40% (Illana et al., 1996).

### TP residues involved in priming activity

Site-directed mutagenesis has been carried out at the TP priming residue Ser232. The change of Ser232 to Thr gives rise to a protein completely inactive in the initiation reaction (Garmendia et al., 1988). Similarly, the change of Ser232 into Cys almost completely abolishes the initiation capacity of the TP mutant, being its initiation capacity about 1% of that of the wild-type TP (Garmendia et al., 1990). These TP mutants interacted in a wild-type manner with both Φ29 DNAP and DNA (Garmendia et al., 1988, 1990). Furthermore, mutation of TP priming-loop residues Leu220 and Ser226 into Pro highly impaired the initiation activity but did not affect either DNA polymerase or DNA-binding, suggesting the implication of these residues in the initiation reaction (Garmendia et al., 1990).

### TP residues involved in DNA-binding

Φ29 TP binds to both single-stranded and double-stranded DNA *in vitro* (Prieto, 1986; Zaballos and Salas, 1989). As mentioned above, the TP domain responsible for non-specific DNA-binding is the N-terminal domain (Zaballos and Salas, 1989). As in many non-sequence specific DNA-binding proteins, TP N-terminal domain basic residues are implicated in its DNA-binding capacity (Holguera et al., 2014).

Viral DNA replication in prokaryotes takes place at specific subcellular locations. In this sense, the use of host organizing structures seems to be essential to provide an appropriate scaffold for viral DNA replication. Φ29 TP localizes at the bacterial nucleoid along the infective cycle, being the N-terminal domain responsible for this localization (Muñoz-Espín et al., 2010). Additionally, parental TP (and therefore TP-DNA) localizes at the bacterial nucleoid, independently of primer TP (Muñoz-Espín et al., 2010). Importantly, the TP N-terminal domain is essential for an efficient viral DNA replication *in vivo* (Muñoz-Espín et al., 2010). To determine the TP residues involved in nucleoid targeting, each basic residue of the TP N-terminal domain was replaced independently by alanine, and the subcellular localization of the resulting proteins fused to YFP was analyzed. Lys27 was the only TP residue that, changed individually, impaired the TP nucleoid localization (Holguera et al., 2014). By using X-Chip techniques, it was shown that wild-type Φ29 TP, but not mutant K27A, binds *B. subtilis* genome *in vivo*, establishing a correlation between nucleoid localization and DNA-binding (Holguera et al., 2014). During the infective cycle both TP and viral DNA polymerase localize at the bacterial nucleoid, being the nucleoid localization of the DNA polymerase dependent on the expression of TP (Muñoz-Espín et al., 2010). The subcellular localization of the viral DNA replication machinery at the bacterial nucleoid has been proposed to serve as a compartmentalization mechanism to make the replication process more efficient, as well as a means of taking advantage of the bacterial chromosome segregation dynamics (Muñoz-Espín et al., 2010). The impact of bacterial chromosome TP binding on host processes such as DNA replication and transcription remains to be investigated.

Interestingly, Φ29 TP localizes at the bacterial nucleoid when expressed in the distantly related bacterium *E. coli*, being the TP N-terminal domain the one responsible for this localization (Muñoz-Espín et al., 2010; Redrejo-Rodríguez et al., 2013). Furthermore, the TP from phage PRD1, which infects *E. coli* among other bacteria, localizes at the *E. coli* nucleoid independently of other viral components. TPs from other phages such as Cp-1, Nf, and GA-1 also localize at the *E. coli* nucleoid, although localization in their host systems remains to be determined (Redrejo-Rodríguez et al., 2013). Altogether, these results suggest that nucleoid localization is a functional property conserved in phage TPs. Importantly, a Nuclear Localization Signal (NLS) has been described in Φ29 TP, as well as in a variety of other TPs such as those from Nf, PRD1, Bam35, and Cp-1 phages (Redrejo-Rodríguez et al., 2012).

### TP residues involved in DNA polymerase-binding

The extensive interactions of Φ29 TP intermediate and priming domains with the DNA polymerase account for the high stability of the heterodimer (Lázaro et al., 1995; Kamtekar et al., 2006). The crystallographic structure of the heterodimer shows that the TP intermediate domain is structurally complementary to the DNA polymerase TPR1 subdomain; this interface has many charged residues that include two salt bridges between TP residues Arg158 and Arg169, and DNA polymerase residues Glu291 and Glu322, respectively (Kamtekar et al., 2006; Figure 2A). In the case of the highly electronegative TP priming domain, the structure shows interactions between TP residues Glu191 and Asp198, and DNA polymerase thumb subdomain residues Lys575 and Lys557, respectively (Figure 2B). In addition, TP residues Gln253 and Tyr250 would interact with DNA polymerase exonuclease domain residue Arg96 through a hydrogen bond and a stacking interaction, respectively (Kamtekar et al., 2006; Figure 2C). In this sense, mutation of DNA polymerase residue Arg96 to alanine was shown to impair the interaction with TP (Rodríguez et al., 2004). Similarly, TP residues Glu252, Gln253, and Arg256 from the C-terminal helix of the priming domain would pack against DNA polymerase TPR2 subdomain residues Leu416, Gly417, and Glu419, respectively (Kamtekar et al., 2006; Figures 2C,D). In fact, by biochemical analysis of TP mutants, TP residues Arg158, Arg169, Glu191, Asp198, Tyr250, Glu252, Gln253, and Arg256 were shown to be involved in the interaction between TP and DNA polymerase (del Prado et al., 2012). Additionally, biochemical studies using both TP and DNA polymerase mutant proteins strongly suggest that TP priming loop residue Glu233 interacts directly with the DNA polymerase palm subdomain residue Lys529 during the first step of TP-DNA replication (del Prado et al., 2013).

### TP interaction with other viral proteins

Apart from the DNA polymerase, Φ29 TP interacts with other viral proteins. By means of *in vitro* chemical crosslinking, it has been shown that Φ29 TP interacts with the viral early protein p1, which is a membrane-associated protein. Based on these results, a model of membrane anchorage of the viral replication machinery mediated by p1 has been proposed (Bravo et al., 2000). In addition, TP interacts with the membrane protein p16.7 *in vitro* (Serna-Rico et al., 2003), presenting another anchoring point to the bacterial membrane. This interaction has also been proposed to facilitate the binding of p16.7 to the displaced strands of the viral genome, favoring their recruitment to the bacterial membrane (Serna-Rico et al., 2003). Mutations introduced at several residues of the TP N-terminal and intermediate domains impaired DNA replication when TP acted simultaneously as primer and parental TP, suggesting that a proper interaction between primer and parental TP is important for origin recognition (Illana et al., 1999; Serna-Rico et al., 2000; del Prado et al., 2012; Holguera et al., 2015).

## Φ29 DNA polymerase

### Processive polymerization coupled to strand displacement: two specific attributes of Φ29 DNA polymerase

Φ29 DNAP is a small (66 kDa) single subunit enzyme, the product of the viral gene 2, characterized as the viral DNA replicase (Blanco and Salas, 1984, 1985b; Salas, 1991), and belonging to the family B (eukaryotic-type) of DNA-dependent DNA polymerases (Blanco and Salas, 1986; Bernad et al., 1987). As any other conventional DNA polymerase, Φ29 DNAP catalyzes the sequential template-directed addition of dNMP units onto the 3′-OH group of a growing DNA chain in a faithful manner as it shows discrimination values of 10^4^–10^6^, and a poor mismatch elongation efficiency (Esteban et al., 1993). Extensive site directed mutagenesis studies in Φ29 DNAP described the function of specific amino acids at motifs YxGG, Dx_2_SLYP, LExE, Kx_3_NSxYG, Tx_2_GR, YxDTDS, and KxY, placed at the C-terminal domain (residues 190–572; polymerization domain) and highly conserved among the eukaryotic DNA polymerases from family B (Blanco and Salas, 1995, 1996; Pérez-Arnaiz et al., 2006, 2009, 2010; Salas and de Vega, 2006; del Prado et al., 2013; Santos et al., 2014). These investigations allowed the identification of the catalytic residues responsible for coordinating the metal ions and the ones acting as ligands of the substrates (DNA, TP, and dNTP).

In contrast to the complexity of other *in vitro* replication systems, efficient synthesis of full-length Φ29 TP-DNA can be accomplished *in vitro* with only the presence of TP and Φ29 DNAP (Blanco and Salas, 1985b). The efficiency of this minimal replication system relies on three unique catalytic features of Φ29 DNAP: (1) ability to initiate DNA replication by using a TP as primer (Salas, 1991), thus bypassing the need for a primase (see below). (2) an extremely high processivity (>70 kb, measured by rolling circle replication, the highest described for a DNA polymerase), allowing replication of the entire genome from a single binding (and priming) event, without the assistance of processivity factors (Blanco et al., 1989); (3) unlike most replicases, Φ29 DNAP efficiently couples DNA polymerization to strand displacement, without the need of helicase-like proteins (Blanco et al., 1989). These three aforementioned exceptional properties are essential to allow the symmetric DNA replication mode of bacteriophage Φ29 mentioned above, by which the two DNA strands are synthesized continuously from both ends of the linear molecule (Blanco et al., 1989). In the case of Φ29 TP-DNA amplification, the single-stranded DNA binding protein p5 and the double-stranded DNA binding protein p6 are essential.

**Figure 3:**
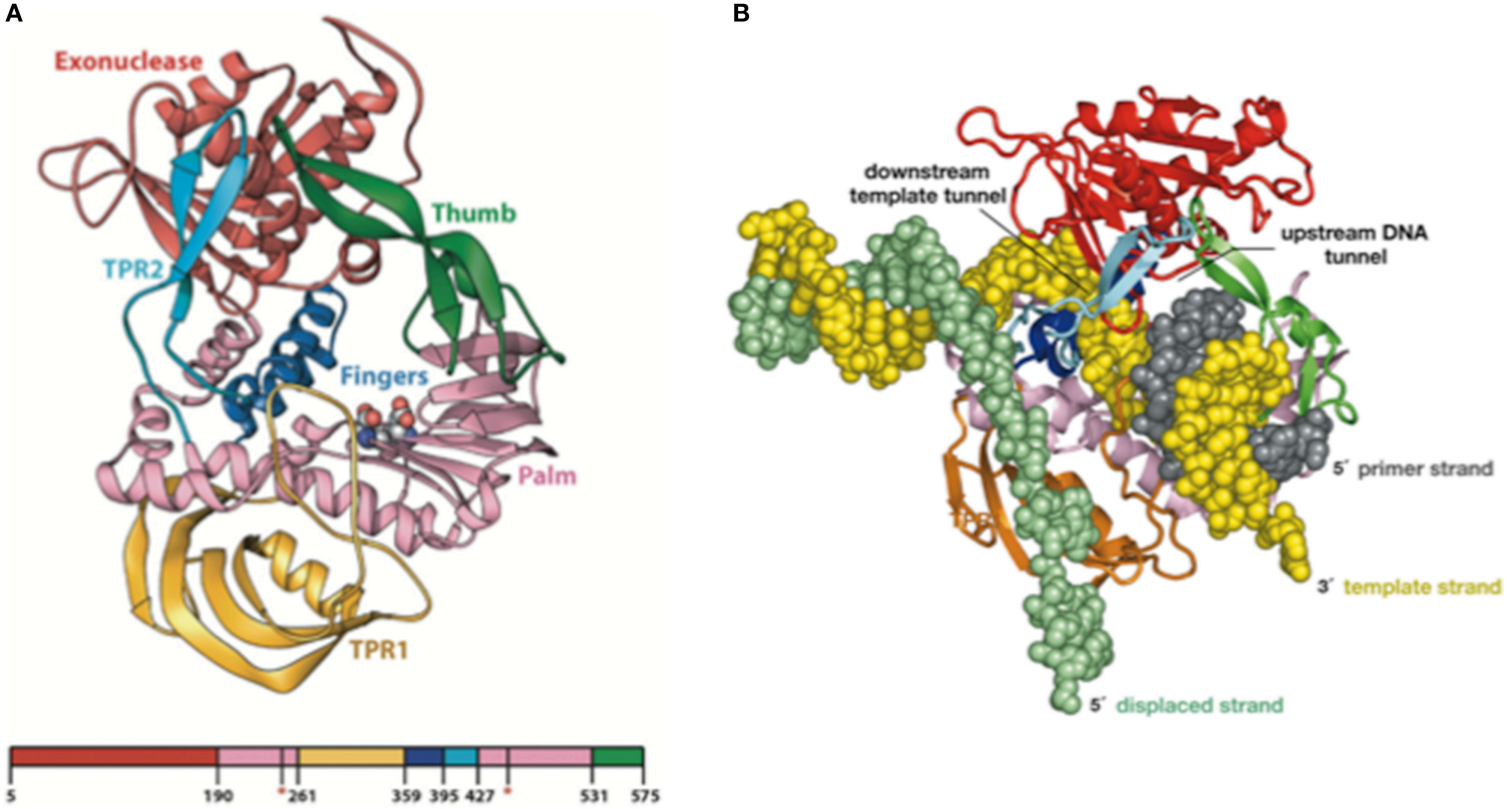
**(A)** Ribbon Representation of the Domain Organization of Φ29 DNAP. The exonuclease domain is shown in red, the palm in pink, TPR1 in gold, the fingers in blue, TPR2 in cyan, and the thumb in green. Asp249 and Asp458, which provide the catalytic carboxylates of the polymerase active site, are shown using space-filling spheres. Reproduced with permission from Kamtekar et al. (2004). **(B)** Modeling processivity and strand displacement in Φ29 DNAP. The TPR2 insertion would contribute to a full encirclement of the DNA substrate, conferring a remarkable processivity, and also acts as a structural barrier, which would force the DNA strands of the parental DNA to diverge (melt). Because Φ29 DNAP translocates after each polymerization cycle, the TPR2 subdomain would act as a wedge to couple polymerization to strand displacement. Reproduced with permission from Rodríguez et al. (2005). Copyright (2005) National Academy of Sciences, U.S.A.

Resolution of the Φ29 DNAP structure, in collaboration with Thomas Seitz's lab (Yale University), gave the insights into these three unique properties of the enzyme, use of TP as primer, processivity, and strand displacement capacity (Kamtekar et al., 2004, 2006). Thus, the Φ29 DNAP structure is formed by an N-terminal exonuclease domain, harboring the 3′–5′ exonuclease active site, and a C-terminal polymerization domain (see Figure 3A) that has the universally conserved palm (containing the catalytic residues as well as DNA ligands), fingers (mainly involved in binding the incoming dNTP), and thumb (containing DNA ligands which confer stability to the primer-terminus) subdomains (Kamtekar et al., 2004). Although *a priori* this bimodular structure would be a common theme among proofreading DNA polymerases, the main structural novelty was the presence in the polymerization domain of Φ29 DNAP of two subdomains called TPR1 and TPR2, specifically present in the protein-primed subgroup of DNA polymerases (Blasco et al., 1990; Dufour et al., 2000). TPR1 is placed at the edge of the palm, while TPR2 contains a β-hairpin and forms with the apex of the thumb subdomain an arch-like structure. Palm, thumb, TPR1, and TPR2 subdomains form doughnut-shaped structure at the polymerization active site that encircles the growing DNA product (Berman et al., 2007), acting as an internal clamp that confers the DNA-binding stability responsible for the inherent processivity of the enzyme (Rodríguez et al., 2005), similar to the sliding clamps used by other replicative polymerases (see Figure 3B). On the other hand, TPR2, palm and fingers subdomains, together with the exonuclease domain, encircle the downstream template strand (Berman et al., 2007), forming a narrow tunnel whose dimensions (~10 Å) do not allow dsDNA binding. This fact forces the unwinding of the downstream dsDNA to allow threading of the template strand through this tunnel to reach the polymerization site, using the same topological mechanism as hexameric helicases to open dsDNA regions (see Figure 3B), providing the structural basis for the strand displacement capacity of Φ29 DNAP (Kamtekar et al., 2004; Rodríguez et al., 2005). The use of optical tweezers has allowed to conclude that the DNA polymerase destabilizes the two nearest base pairs of the fork by maintaining a sharp bending of the template and the complementary strands at a closed fork junction (Morin et al., 2012). Therefore, the polymerase, instead of behaving as a “passive” unwinding motor that would imply that translocation of the protein traps transient unwinding fluctuations of the fork, behaves as an “active” motor, actively destabilizing the duplex DNA at the junction.

### On the translocation mechanism of Φ29 DNA polymerase

**Figure 4:**
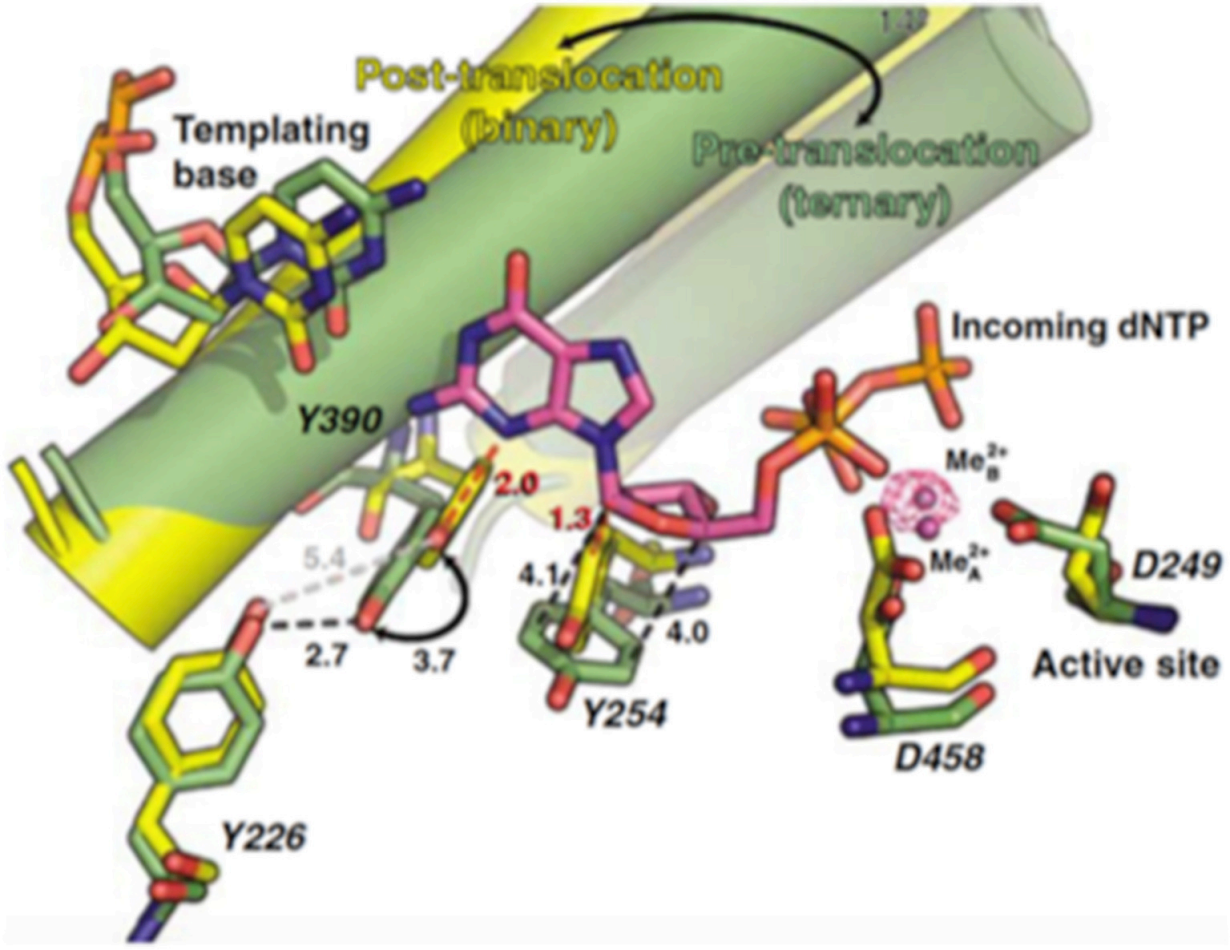
**Comparison of the binary (yellow) and ternary (green) complex structures of Φ29 DNAP**. The mechanistically significant amino acid movements are indicated. Reproduced with permission from Berman et al. (2007).

As any other replicative DNA polymerase, after inserting a dNMP, Φ29 DNAP has to translocate the growing DNA one position backwards to allow the next insertion step to occur, a process called translocation. The structures of the binary and ternary complexes of Φ29 DNAP provided a structural basis for comprehending the mechanism of translocation (Berman et al., 2007). The dNTP insertion site is initially occupied by the aromatic ring of two conserved residues, Tyr390 (from the fingers subdomain) and Tyr254 (from the palm subdomain; see Figure 4). Once the incoming nucleotide gains access and binds at the polymerization active site it triggers a 14° rotation of the fingers subdomain toward the polymerization active site, going from an open to a closed state and allowing electropositively charged residues from the fingers subdomain to bind the α- and β-phosphates of the dNTP. Closing of the fingers provokes Tyr390 and Tyr254 to abandon the nucleotide insertion site to form part of the nascent base pair binding pocket, allowing the base moiety of the incoming nucleotide to form a Watson-Crick base pair with the templating nucleotide, whereas the deoxyribose ring stacks on the phenolic group of Tyr254. Once the phosphoester bond formation between the α-phosphate of the incoming dNTP and the OH-group of the priming nucleotide has taken place (pre-translocation state), the pyrophosphate produced leaves the DNA polymerase, breaking the electrostatic crosslink that kept the fingers subdomain in the closed state. Concomitantly to the fingers opening, residues Tyr254 and Tyr390 move back into the nucleotide insertion site, and the nascent base pair translocates backwards one position (post-translocation state; Berman et al., 2007). This translocation allows the 3′ OH-group of the newly added nucleotide to be in a competent position to prime the following nucleotide insertion event. Direct observation of translocation in individual Φ29 DNAP complexes monitored with single nucleotide resolution and using the hemolysin nanopore, has allowed to conclude that Φ29 DNAP translocation occurs discretely from the pre-translocation state to the post-translocation state, driven by Brownian thermal motion (Dahl et al., 2012). Although nucleotide does not drive translocation, the fluctuation of the binary complexes between the pre-translocation and post-translocation states is rectified to the post-translocation state by the binding of complementary dNTP. The movement from the open, post-translocation state, to the closed pre-translocation state most probably reflects an equilibrium between the fingers-open and fingers-closed states to relieve the steric clash of the primer-terminus with residues Tyr254 and Tyr390 (see above), which occlude the nucleotide insertion site when the fingers are open (Dahl et al., 2012).

### The degradative reactions catalyzed by Φ29 DNA polymerase: The pyrophosphorolysis and the 3′–5′ exonuclease activity

In addition to the synthetic activities described above, ϕ29 DNAP catalyzes two degradative reactions:

1. Pyrophosphorolysis. Φ29 DNAP possesses an inorganic pyrophosphate-dependent degradative activity, pyrophosphorolysis (Blasco et al., 1991). This activity, whose optimal substrate is a duplex DNA with a protruding 5′ single strand, can be considered as the reversal of polymerization as it acts in the 3′–5′ direction releasing free dNTPs by addition of PPi as substrate, in the presence of divalent metal ions, probably playing some role in fidelity (Blasco et al., 1991). The fact that Φ29 DNAP mutants at the catalytic amino acid residues involved in the DNA polymerization activity were also deficient in the pyrophosphorolytic activity indicated that both activities share a common polymerization active site (Blasco et al., 1991; Santos et al., 2014).
2. 3′–5′ exonuclease. A reaction found in the N-terminal domain of the polymerase, and that requires two divalent metal ions to release dNMP units from the 3′ end of a DNA strand at a catalytic rate of 500 s^−1^ (Esteban et al., 1994). As in other replicases, the 3′–5′ exonuclease of Φ29 DNAP proofreads DNA insertion errors, as it degrades preferentially mismatched primer termini (Blanco and Salas, 1985a; Garmendia et al., 1992).

**Figure 5:**
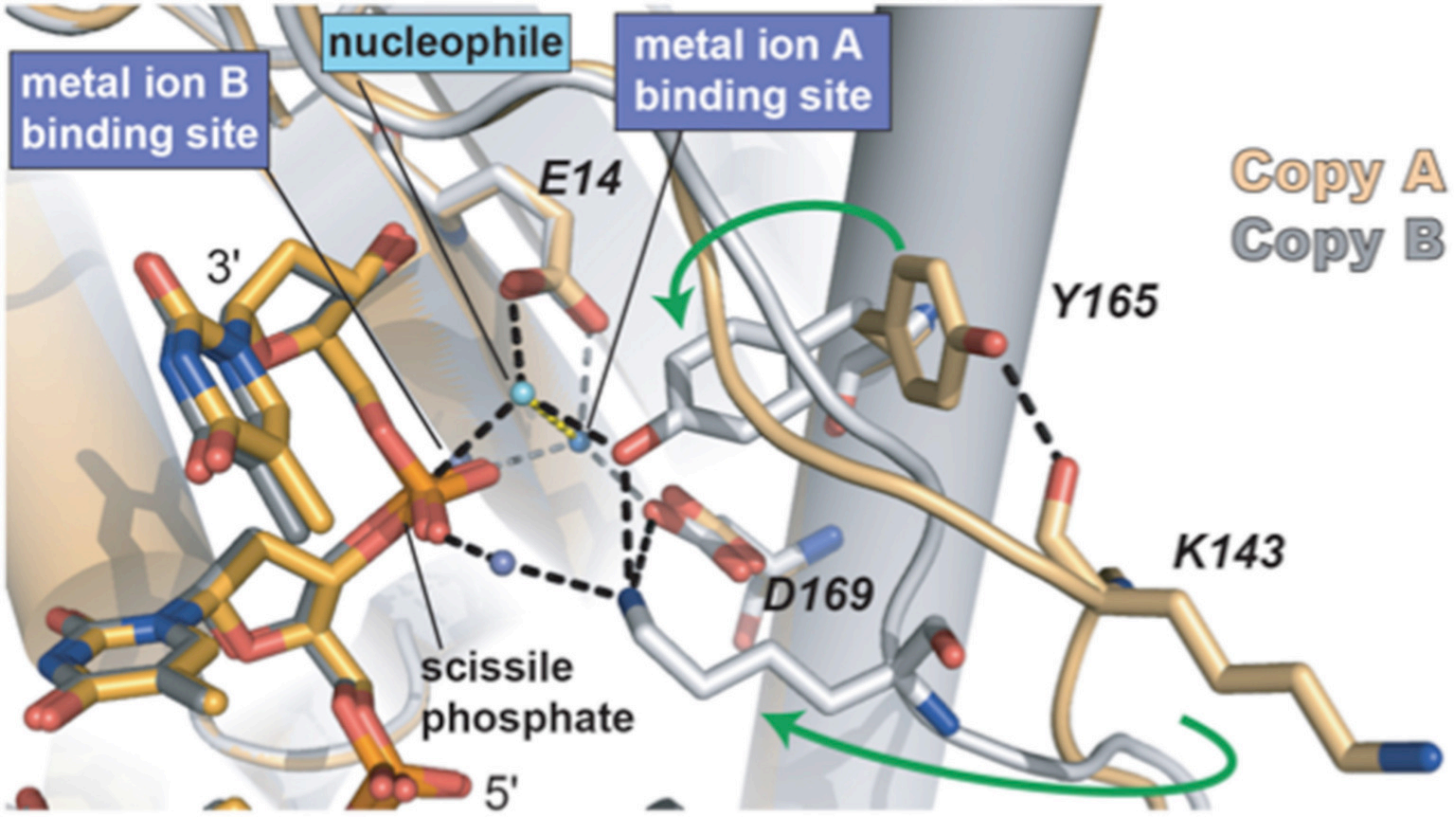
**The two conformations of the exonuclease active site of Φ29 DNAP**. The Lys143, Tyr165, and two of the catalytic aspartates are shown in stick representation. Green arrows indicate the movement of Tyr165 and Lys143 from the open conformation to the closed conformation. The black dashed lines represent the observed hydrogen bonds between Lys143 and Tyr165 with each other and with other parts of the active site. The interactions between the waters in the metal binding sites and the protein are represented as gray dashes. Most of the interactions that the water in the metal in **B** site would be making with the protein are missing due to the D12A/D66A mutations in the polymerase used in these crystallographic studies. Reproduced with permission from Berman et al. (2007).

Sequence alignments and extensive site directed mutagenesis studies carried out during the last three decades in Φ29 DNAP have been pioneer in the identification and role of the catalytic and ssDNA ligand residues responsible for the 3′–5′ exonuclease (reviewed in Blanco and Salas, 1995, 1996). The presence of homologous residues among distantly related DNA-dependent DNA polymerases allowed us to propose the evolutionary conservation of 3′–5′ exonuclease active site (Bernad et al., 1989) in the proofreading DNA polymerases. Thus, the exonuclease active site, located at the N-terminal domain (residues 1–189; exonuclease domain, see Figure 3), is formed by three conserved N-terminal amino acid motifs, ExoI, ExoII, and ExoIII, that contain four carboxylate groups (Asp12, Glu14, Asp66, and Asp169 in Φ29 DNAP) that coordinate two metal ions, and one tyrosine residue (Tyr165 in Φ29 DNAP) that orients the attacking water molecule (Bernad et al., 1989). Moreover, these analyses allowed the identification of a new motif (Kx_2_*h*xA), specifically conserved in family B DNA polymerases and whose lysine residue (Φ29 DNAP Lys143) plays an auxiliary role in catalysis (de Vega et al., 1997), stabilizing the catalytic Asp169 of the Exo III motif (Berman et al., 2007). Crystallographic resolution of Φ29 DNAP with a ssDNA at the exonuclease active site demonstrated the existence of two stable conformations at the exonuclease active site of family B DNA polymerases (see Figure 5), as previously suggested from comparisons of T4 and RB69 DNA polymerase exonuclease structures with the *E. coli* DNA polymerase I Klenow fragment exonuclease structure (Beese and Steitz, 1991; Wang et al., 1996, 2004). In one conformation, the tyrosine from the Exo III motif (Tyr165 in Φ29 DNAP) is solvent exposed, whereas in the other conformation, it contacts with the scissile phosphate through the nucleophile while conserved lysine from motif Kx_2_*h*xA (Φ29 DNAP Lys143) stabilizes the catalytic aspartate of the Exo III motif (Φ29 DNAP Asp169), consistent with the previous biochemical results (de Vega et al., 1997). The latter conformation seems to be the more chemically and biologically relevant complex for exonuclease activity. The two conformations observed suggest that the movement of the conserved tyrosine and lysine residues into the active site sets up the active site for the exonucleolysis reaction in the family B DNA polymerases.

A tight and fine-tuned coordination between the polymerization and exonucleolytic cycles should take place to allow a productive and faithful replication. Previous studies showed that Φ29 DNAP proofreads the misinserted nucleotides intramolecularly (de Vega et al., 1999). This fact implies that the DNA polymerase transfers the mismatched 3′-teminus to the 3′–5′ exonuclease active site for erroneous dNMP release without dissociating from the DNA. Comparison of DNA polymerase structures of RB69 DNA polymerase in polymerization and editing modes showed that the primer-terminus switches between both active sites by the rotation of a top microdomain of the thumb subdomain (Shamoo and Steitz, 1999; Franklin et al., 2001). However, in the 3D resolution of Φ29 DNAP structure the thumb subdomain has an unusual structure mainly constituted by a static long β-hairpin that does not rotate upon DNA binding (Kamtekar et al., 2004; Berman et al., 2007, see Figure 3). In addition, the blockage of the thumb movements by introducing a disulfide bond between the tips of the TPR2 and thumb subdomains had not effect in the partitioning of the primer-terminus between the polymerization and editing active sites (Rodríguez et al., 2009), a result that led us to suggest that in Φ29 DNAP the primer-terminus switches between both active sites by a passive diffusion mechanism. In this sense, the recent use of single-molecule manipulation method has made possible the study of the dynamics of the partitioning mechanism by applying different tension to a processive single Φ29 DNAP-DNA complex (Ibarra et al., 2009). Thus, the application of mechanical force to the template causes the gradual intramolecular switch of the primer between the active sites of the protein by decreasing the affinity of the polymerization active site for the template strand with the further disruption of the dsDNA primer-template structure that provokes a fraying of 4–5 bp of dsDNA, allowing primer-terminus to reach the exonuclease active site intramolecularly (Ibarra et al., 2009), supporting the passive diffusion mechanism. The energetically unfavorable gradual melting of 4–5 bp of dsDNA should be progressively balanced by the establishment of new and specific interactions with DNA ligands of the thumb subdomain (Pérez-Arnaiz et al., 2006). Such contacts would also guide the primer-terminus to interact with ssDNA ligands of the exonuclease domain that stabilize the primer-terminus at the exonuclease site (de Vega et al., 1996, 1998b; Kamtekar et al., 2004; Pérez-Arnaiz et al., 2006; Rodríguez et al., 2009).

Recent development of a single-molecule approach using a nanoscale pore has allowed to conclude that transfer of the primer strand from the polymerase to the exonuclease site takes place before translocation, the pre-translocation state being therefore the branchpoint between the DNA synthesis and editing pathways (Dahl et al., 2014). Once the 3′ terminal nucleotide is released, the primer-terminus goes back to the polymerase site and pairs with the template strand in the post-translocation state being poised to bind the incoming dNTP and resume DNA synthesis (Dahl et al., 2014).

### Biotechnological applications of Φ29 DNA polymerase

The two distinctive features of Φ29 DNAP, high processivity, and strand displacement capacity, together with a remarkably faithful replication, contributed by a high nucleotide insertion fidelity, and an intrinsic proofreading activity, led to the development of isothermal multiple displacement amplification (MDA) currently exploited (Dean et al., 2001, 2002). These amplification methods based on Φ29 DNAP show two main advantages respect to classical PCR DNA amplification: first, the use of random hexamer primers eliminates the previous sequence information requirement allowing the amplification of any DNA molecule, and second, the products of the amplification reaction can be much larger than those obtained by classical PCR. In addition, the capacity displayed by Φ29 DNAP to use circular multiply primed ssDNA templates gave rise to the development of the multiply primed rolling circle amplification of circular DNAs of variable size (Dean et al., 2001). This technology has been successfully exploited to amplify and detect circular viral genomes (Johne et al., 2009), to genotype single nucleotide polymorphisms (Qi et al., 2001), to analyze the genome of non-cultivable viruses (Johne et al., 2009), to detect and identify circular plasmids in zoonotic pathogens (Xu et al., 2008), and to describe new metagenomes (López-Bueno et al., 2009). Recently, we have been able to improve isothermal MDA by making new variants of Φ29 DNAP (de Vega et al., 2010). Thus, we have fused DNA binding domains (Helix-hairpin-Helix) to the C-terminus of the polymerase increasing the DNA binding ability of the enzyme without compromising its replication rate. As a result, the new variants display an improved DNA amplification efficiency on both circular plasmids and genomic DNA and are the only Φ29 DNAP variants with enhanced amplification performance so far.

## Initiation opposite an internal templating nucleotide: A smart solution to preserve the fidelity during initiation

The Φ29 TP/DNAP heterodimer recognizes the replication origins at the genome ends (see Figure 1). Such origins are constituted by specific sequences as well as by the parental TP, the major signal in the template for recognition, a fact that suggests that the heterodimer is recruited to the origin through interactions with the parental TP. The use of heterologous systems in which DNA polymerase, primer TP, and TP-DNA were from the Φ29 and Nf related phages allowed us to infer specific contacts between the DNA polymerase and the parental TP, as the initiation only occurred when the polymerase and the TP-DNA were from the same phage (González-Huici et al., 2000b; Pérez-Arnáiz et al., 2007). In addition, the presence of mutations in the intermediate domain of both the parental and primer TPs precluded DNA replication, suggesting also a role for the primer TP in the specific recognition of the replication origins (Illana et al., 1998; Serna-Rico et al., 2000; del Prado et al., 2012).

**Figure 6:**
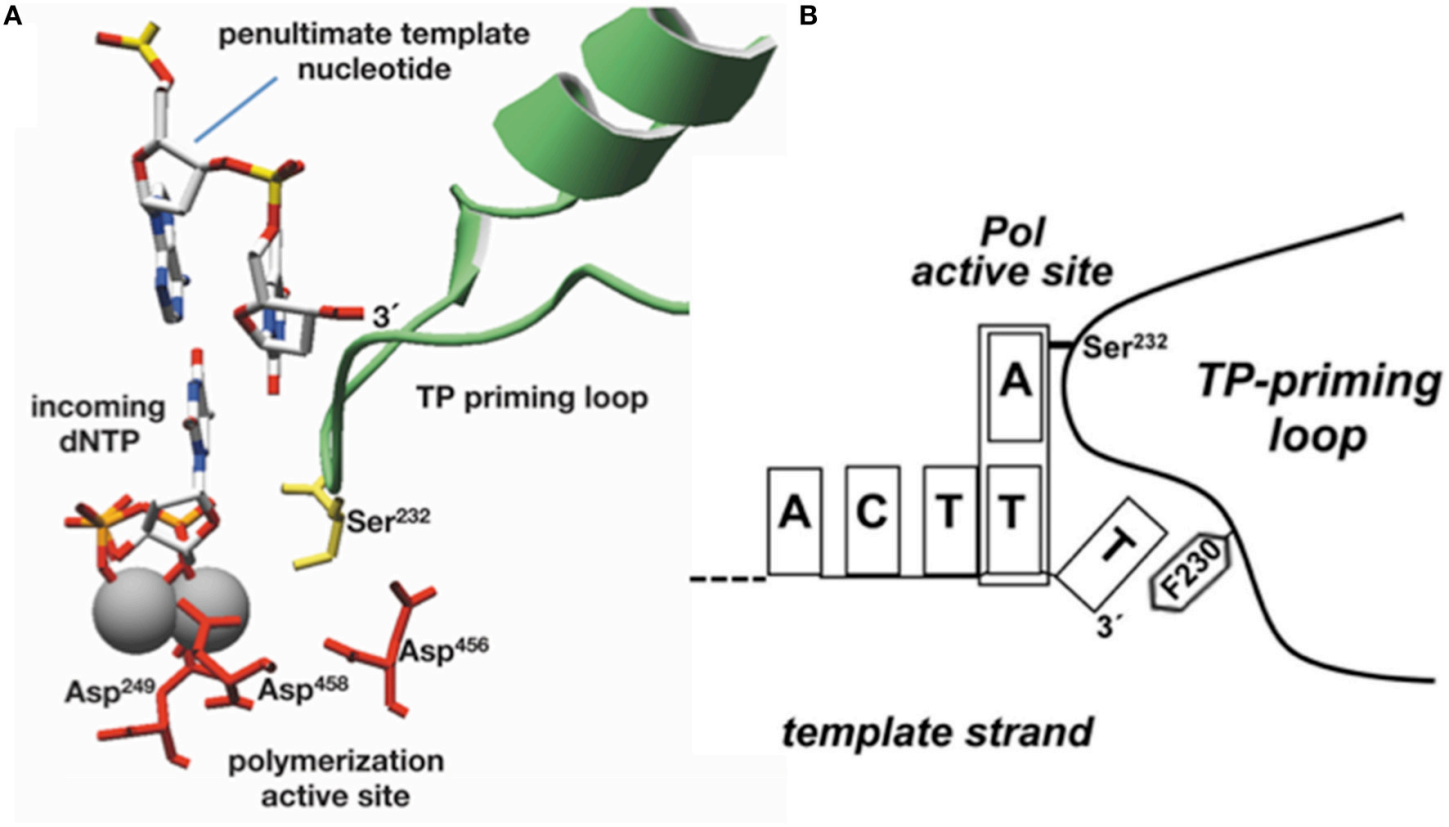
**Modeling the initiation reaction. (A)** Placement of TP priming residue Ser232 (in lemon green) and penultimate template nucleotide at the Φ29 DNAP active site during initiation of Φ29-DNA replication (catalytic aspartates and Mg^2+^ ions are shown in red and gray, respectively). Reproduced from de Vega and Salas (2011). **(B)** Schematic representation of the placement of the 3′ end of the template strand at the active site of Φ29 DNA polymerase. The templating nucleotide and the aromatic residue of the priming loop are indicated. This research was originally published in The Journal of Biological Chemistry. del Prado et al. (2015). Copyright©2015, by the American Society for Biochemistry and Molecular Biology.

**Figure 7:**
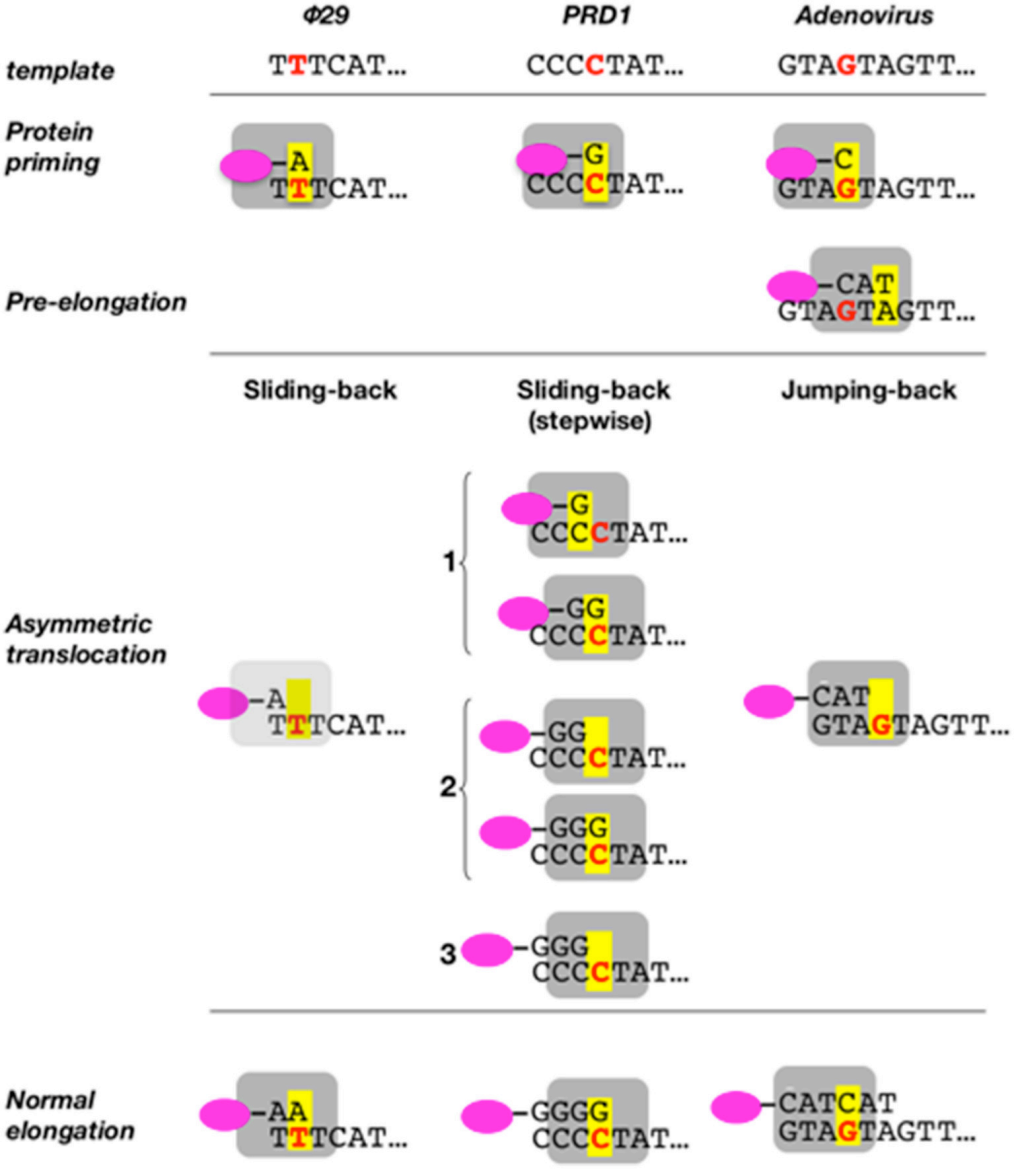
**Sliding-back (jumping-back) model for the transition from initiation to elongation**. TP is represented as a pink oval and DNA polymerase as a gray square. The internal template nucleotide that directs the insertion of the initial nucleotide is shown in bold red letter. Yellow box represents the catalytic active site of the DNA polymerase. Reproduced from de Vega and Salas (2011).

As already indicated, the DNA ends of Φ29 have a repetition of three nucleotides (3′-TTT…. 5′). Once the replication origins are specifically recognized by the TP/DNA polymerase heterodimer (Blanco et al., 1987; Freire et al., 1996; González-Huici et al., 2000a,b; Pérez-Arnáiz et al., 2007), the DNA polymerase catalyzes the formation of a phosphoester bond between the initial dAMP and the hydroxyl group of Ser232 of the TP. Therefore, during the initiation reaction, the priming Ser232 of the TP is placed at the catalytic site of the DNA polymerase to attack nucleophilically the α-phosphate of the initial dAMP which is inserted opposite the 3′ second nucleotide of the template strand (Méndez et al., 1992, see Figure 6A). This reaction is carried out by the catalytic residues responsible for canonical polymerization (Blanco and Salas, 1995, 1996). The initiation reaction implies that the 3′ end of the template strand should enter deep into the catalytic site of the DNA polymerase to place the penultimate 3′ dTMP of the template strand at the catalytic site (see Figures 6A,B). The interchanging of the priming domains of the related Φ29 and Nf TPs, allowed us to conclude that this domain is the one responsible for dictating the internal 3′ nucleotide used as template during initiation, the 2nd and 3rd in Φ29 and Nf DNA, respectively (Longás et al., 2008). Recently, we have shown that the aromatic residue Phe230 of the Φ29 TP priming loop is the one responsible for positioning the penultimate nucleotide at the polymerization site to direct insertion of the initial dAMP during the initiation reaction, most probably by interacting with the 3′ terminal base, limiting the internalization of the template strand (see del Prado et al., 2015; Figure 6B). To perform TP-DNA full-length synthesis, the TP-dAMP initiation product translocates backwards one position to recover the template information corresponding to the first 3′-T, the so-called sliding-back mechanism that requires a terminal repetition of 2 bp. This reiteration permits, prior to DNA elongation, the asymmetric translocation of the initiation product, TP-dAMP, to be paired with the first T residue (Méndez et al., 1992) (see scheme in Figure 7).

We have shown that the sliding-back, or variations of it, is a mechanism shared by the protein-priming systems to restore full-length DNA. In the case of the Φ29-related phage GA-1, initiation takes place at the 3′ second nucleotide of the template (3′-TTT) (Illana et al., 1996). The Φ29-related phage Nf and the *S. pneumoniae* phage Cp-1 initiate opposite the 3′ third nucleotide of their terminal repetition (3′-TTT) (Martín et al., 1996; Longás et al., 2008), whereas the *E. coli* phage PRD1 initiates at the fourth nucleotide (3′-CCCC) (Caldentey et al., 1993), being required two and three consecutive sliding-back steps, respectively, to recover the information of the DNA termini (stepwise sliding-back). The case of adenovirus is a little more complex as its genome ends have a duplication of the sequence GTA (3′-GTAGTA). In this virus, the 3′ fourth to sixth template positions guides the formation of the TP-CAT initiation product that jumps back to pair with the terminal GTA, a variation of the sliding-back mechanism called jumping-back (King and van der Vliet, 1994) (see scheme in Figure 7).

What is the rationale of the sliding-back mechanism? Φ29 protein-primed initiation is an unfaithful reaction with a nucleotide insertion discrimination factor about 10^2^. In addition, the 3′–5′ exonucleae activity of Φ29 DNAP cannot release a wrong nucleotide that had been added during the initiation reaction (Esteban et al., 1993). Therefore, the sliding-back mechanism could guarantee the fidelity during the initiation stage through different base pairing checking steps before further elongation of the TP-dNMP complex occurs (Méndez et al., 1992; King and van der Vliet, 1994). Thus, an erroneous TP-dNMP complex will not pair with the terminal 3′-T of the template after the sliding-back, hindering its further elongation. In addition, if an incorrect TP-dNMP product were elongated the resulting TP-DNA molecule could not be used as a template in the next replication round, as the 3′ terminus of the template strand would not include the required nucleotide reiteration. The presence of sequence repetitions at the ends of other TP-containing genomes allows to surmise that the sliding-back type of mechanism could be a common feature of protein-primed replication systems (Méndez et al., 1992).

## Transition from protein-primed to DNA-primed replication

**Figure 8:**
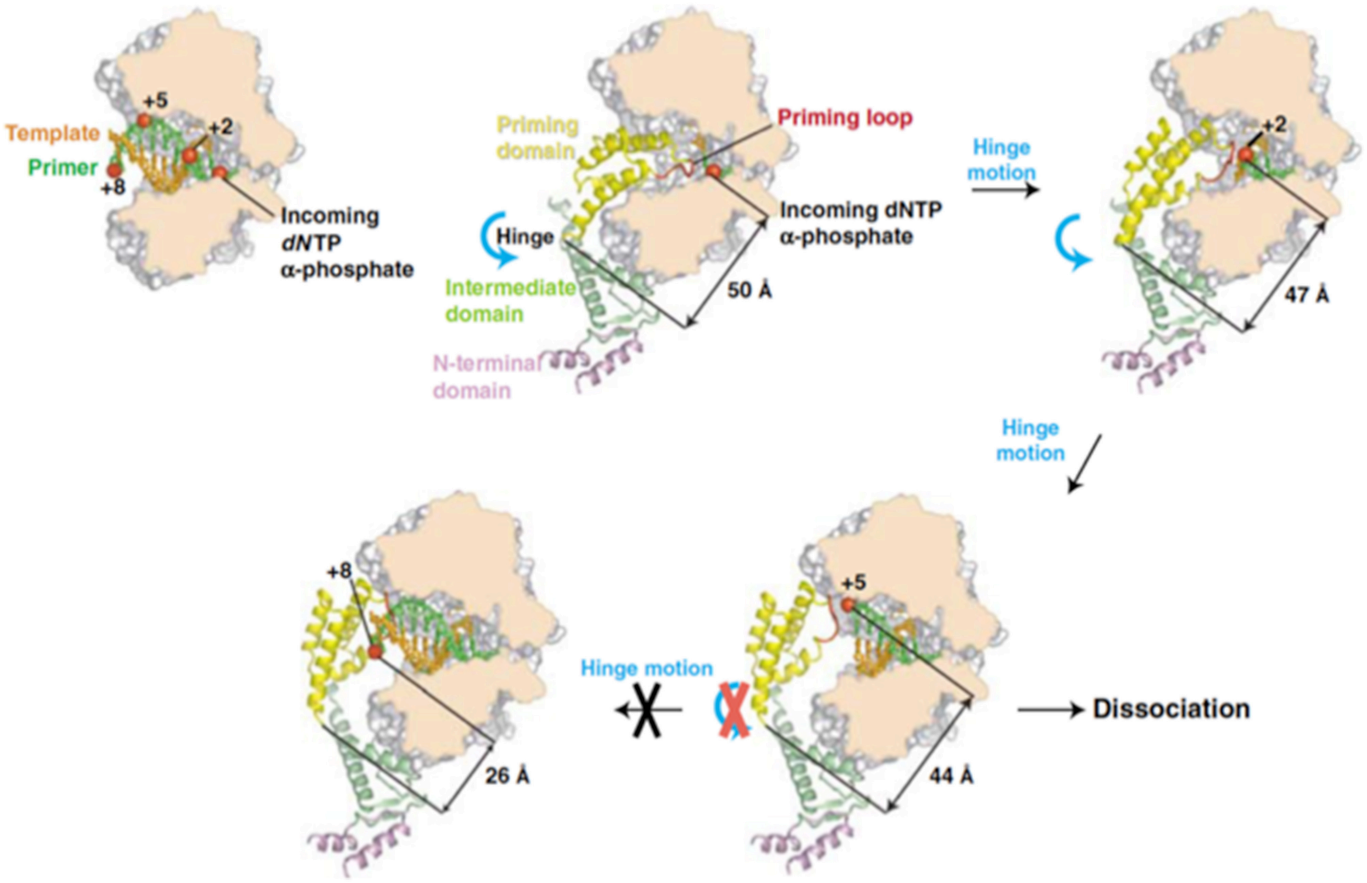
**A model for the transition from initiation of replication to elongation**. Reproduced with permission from Kamtekar et al. (2006).

Previous biochemical studies showed that once the initiation reaction has taken place the polymerase incorporates the next 4 nucleotides to the TP-dAMP product while is still complexed with the primer TP (initiation mode), goes through some structural changes during insertion of the sixth to ninth nucleotide (transition mode) and finally dissociates from the primer TP once the tenth nucleotide is added to the growing strand (elongation mode) (Méndez et al., 1997). Resolution of the Φ29 DNAP/TP complex has given the insights on the transition mechanism, explaining how the polymerase can insert up to nine nucleotides while complexed to the TP (Kamtekar et al., 2006). The transition stage relies on a different strength interaction of the TP priming and intermediate domains with the DNA polymerase (Pérez-Arnáiz et al., 2007). On the one hand, the TP intermediate domain remains in a fixed orientation on the polymerase during insertion of 6–7 nucleotides by means of stable contacts with the TPR1 subdomain. During this stage the weakness of the interaction between the DNA polymerase and the TP priming domain allows the latter to rotate as the DNA is synthesized. The rotation of the TP priming domain with respect to the fixed TP intermediate domain is possible due to the flexibility of the hinge region that connects both domains. Once 6–7 nucleotides have been added, the proximity of the priming Ser to the hinge region would impede a further priming domain rotation, causing heterodimer dissociation (Kamtekar et al., 2006; see Figure 8).

## Φ29 protein p5, the viral single-stranded DNA binding protein

### Structural and functional characteristics

Single-stranded DNA-binding proteins (SSBs) are common in all three branches of organisms and in viruses and bind with high affinity to single-stranded (ss) DNA, playing essential roles as accessory proteins in DNA replication, recombination, and repair processes that entail the exposure of ssDNA. SSBs usually bind non-specifically to DNA and can saturate long stretches of ssDNA, thus providing protection against nuclease attack, and preventing the formation of secondary structures (Chase and Williams, 1986; Kur et al., 2005). Furthermore, SSB proteins are involved in specific interactions with several proteins that play important roles in nucleic acids metabolism (Shereda et al., 2008). As a result of these properties, SSBs increase the efficiency and fidelity of a number of DNA amplification methods (Rapley, 1994; Perales et al., 2003; Inoue et al., 2006; Mikawa et al., 2009; Ducani et al., 2014).

From a structural viewpoint, SSBs exist as monomeric or multimeric proteins and, with few exceptions, they share a structural domain named OB-fold (oligonucleotide/oligosaccharide binding-fold) involved in nucleic acid recognition (Theobald et al., 2003; Savvides et al., 2004). The OB-fold structural domain consists in a close or semi-open beta barrel made out of five-stranded β-strands and a α-helix, commonly between the third and four strands (Murzin, 1993).

**Figure 9:**
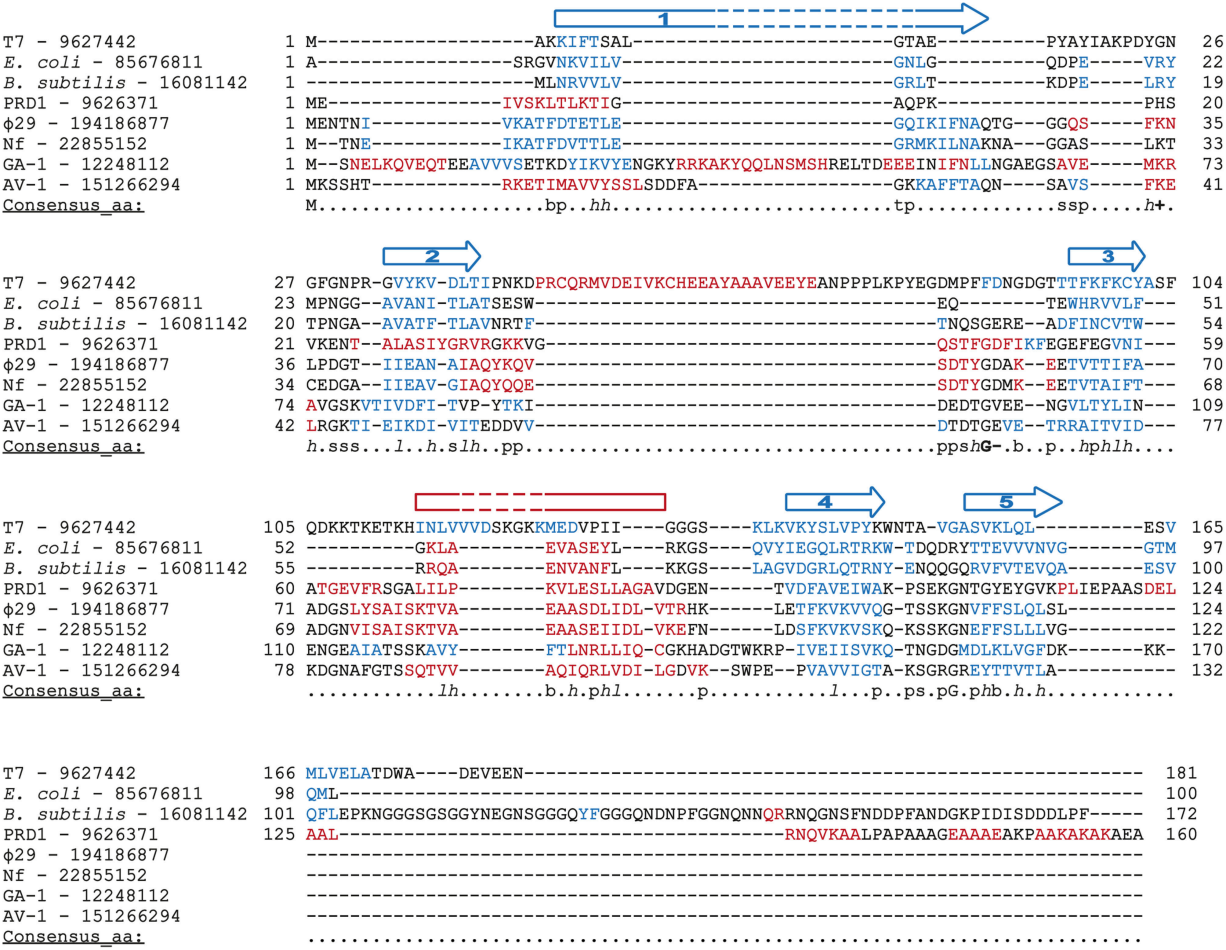
**Multiple sequence alignment of diverse SSBs from prokaryotic origin**. Source and GenBank identification number (GI) of each protein is indicated. Alignment was made with Promals3D (Pei et al., 2008), based secondary structure predictions and the crystal structure of *E. coli* and T7 SSBs (1SRU and 1JE5, respectively, in Protein Data Bank). The protein sequences are colored according to actual or predicted secondary structures (red: alpha-helix, blue: beta-strand). Also, the consensus five beta-strands and the alpha-helix that correspond with a common OB-fold are depicted above the sequences. Note that in the case of T7 SSB the α-helix is between the second and third strands. The last line in each block (Consensus_aa) shows consensus amino acid sequence as follows: conserved amino acids are in uppercase letters; aliphatic (I, V, L): l; aromatic (Y, H, W, F): @; hydrophobic (W, F, Y, M, L, I, V, A, C, T, H): h; alcohol (S, T): o; polar residues (D, E, H, K, N, Q, R, S, T): p; tiny (A, G, C, S): t; small (A, G, C, S, V, N, D, T, P): s; bulky residues (E, F, I, K, L, M, Q, R, W, Y): b; positively charged (K, R, H): +; negatively charged (D, E): −; charged (D, E, K, R, H): c.

Φ29 protein p5 is a single-stranded DNA binding protein (Martin et al., 1989) that protects DNA from nucleases (Martin et al., 1989) and prevents unproductive binding of Φ29 DNAP to ssDNA generated during replication (Gutiérrez et al., 1991). Φ29 SSB has high sequence similarity with SSBs from the related podoviruses Nf and GA-1, although Φ29 and Nf are monomeric in solution, whereas GA-1 SSB is hexameric (Soengas et al., 1995; Gascón et al., 2000a), by means of a N-terminal additional motif (Gascón et al., 2002). Podoviral SSBs share some key hydrophobic residues with unrelated viral SSBs (Gutiérrez et al., 1991) and, indeed, they may also share the SSBs common OB-fold, as found by secondary structure prediction and multiple sequence alignment (Figure 9). In agreement with this predicted protein folding, previous circular dichroism spectra indicated that Φ29 SSB is largely made up of β-strands (Soengas et al., 1997a).

The interaction of Φ29 SSB with ssDNA is consistent with a moderate cooperative binding to 3–4 nt per molecule, not impaired by ionic conditions (Soengas et al., 1994). Detailed analysis of intrinsic tyrosine fluorescence quenching upon binding to ssDNA and site-directed mutagenesis indicated that Tyr50, Tyr57, and Tyr76 play essential role in complex formation with DNA (Soengas, 1996; Soengas et al., 1997b).

As other SSBs, Φ29, Nf, and GA-1 SSBs, are able to unwind duplex DNA (Soengas et al., 1995; Gascón et al., 2000b), suggesting that they can favor DNA replication by unwinding the secondary structures formed in the ssDNA produced during genome replication. However, although all the three SSBs increase DNA replication efficiency (Martin et al., 1989), only Φ29 SSB enhances the replication rate of the DNA polymerase, especially when strand displacement is impaired, although it does not seem to have specificity for its cognate DNA polymerase (Soengas et al., 1995; Gascón et al., 2000b). Therefore, rather than the interaction of the SSB and its own DNA polymerase, improvement of the replication rate by Φ29 SSB is mediated by its dynamic dissociation from the nucleoprotein complexes ahead the polymerase, in agreement with its relative low intrinsic binding constant (Soengas et al., 1994; Gascón et al., 2000a).

### Biological role

SSBs are required in stoichiometric quantities with respect to the template rather than in catalytic amounts. Accordingly, Φ29 SSB is required in high amounts for *in vitro* Φ29 genome amplification (Blanco et al., 1994) and it is an extremely abundant protein in the infected *B. subtilis* cells (~700,000 molecules per cell, Martin et al., 1989). Early genetic characterization of Φ29 mutants allowed the mapping of temperature-sensitive mutants in gene 5 (Mellado et al., 1976). Those mutants had a strong impairment in DNA synthesis (Talavera et al., 1972), indicating an essential role in replication of the viral genome that, as mentioned above, was subsequently demonstrated thanks to the *in vitro* characterization of TP-DNA replication.

Strikingly, recent isolation of a non-sense mutant in gene 5 that only had a 20% reduction in viral yield (Tone et al., 2012), suggested that Φ29 SSB might be dispensable for viral replication, although it seems to be required in a temperature dependent fashion. These results led the authors to speculate that a host SSB could be able to partially complement the absence of viral SSB at permissive temperatures. However, the molecular mechanism of this possible temperature-dependent role of Φ29 SB remains unclear.

## A histone-like protein encoded by bacteriophage Φ29

### Structural characteristics and DNA binding mechanism

The viral protein p6 is a DNA binding protein (DBP) involved both in DNA replication, activating the protein-primed initiation step, and transcriptional control, modulating the early-late switch (for a detailed review see González-Huici et al., 2004a). This pleiotropic effect is consequence of its role as an architectural protein that organizes and compacts the viral genome, analogously to eukaryotic histones (Serrano et al., 1994).

Structure-function work on p6 indicated that the N-terminal region of the protein plays a role in both DNA binding (Otero et al., 1990) and dimer formation (Abril et al., 2000). By site-directed mutagenesis, it could be disclosed that residues Ile8 and Val44 are directly involved in protein dimer formation (Abril et al., 2000, 2002), whereas Lys2, Lys10 and, especially, Arg6, are essential for DNA binding *in vitro* and viral DNA synthesis *in vivo* (Bravo et al., 1994b; Freire et al., 1994).

According to footprinting assays (Prieto et al., 1988; Serrano et al., 1990), p6 binding to DNA gives rise to a nucleoprotein complex formed by a repeated motif of p6 dimers bound to a 24 bp DNA segment. Thus, a protein monomer would contact and bend the DNA every 12 bp, suggesting a model in which the DNA would wrap around a multimeric core of protein p6, forming a right-handed superhelix that comprises around 63 bp per turn (Serrano et al., 1990, 1993a,b). As a consequence of DNA wrapping, this nucleoprotein complexes show a remarkable reduction in length with respect to naked DNA, between 4.2- to 6.5-fold (Serrano et al., 1993a; Gutiérrez et al., 1994).

*In vivo*, p6 is able to discriminate between bacterial and viral DNA by their different superhelicity (González-Huici et al., 2004b). Thus, p6 is able to restrain positive supercoiling of the DNA *in vitro* (Prieto et al., 1988; Serrano et al., 1993b) and binds all along Φ29 DNA *in vivo* with a much higher affinity than for plasmid DNA, although binding to plasmid DNA is enhanced by decreasing the negative supercoiling (González-Huici et al., 2004c). Thus, the presumably lower negative superhelicity of Φ29 DNA respect to host chromosome likely makes the viral genome an appropriate target for the binding of p6 (Serrano et al., 1994; González-Huici et al., 2004b). Interestingly, the preferential binding of Φ29 p6 to the lower negatively supercoiled viral genome seems to be quite specific, since GA-1 p6, which has a highly conserved sequence (58% similarity, 39% identity), does not show this binding pattern (Freire et al., 1996) and accordingly, GA-1 p6 complex with Φ29 DNA is not functional (Alcorlo et al., 2007).

Moreover, p6 has a binding specificity to the ends of the Φ29 linear genome, which has a key role in the initiation step of replication (see below). This binding occurs at recognition regions that were mapped between positions 62–125 from the right end, and between positions 46–68 from the left one (Serrano et al., 1989). However, p6 does not recognize a sequence signal, but rather a sequence-dependent bendability pattern present in the recognition sites that act as a nucleation site for protein p6/DNA complex formation (Serrano et al., 1993a; González-Huici et al., 2004b).

### Functional implications of p6 nucleoprotein complex

Protein p6 is essential for Φ29 DNA replication *in vivo* (Carrascosa et al., 1976; Bravo et al., 1994b). *In vitro*, p6 stimulates initiation as well as the transition to elongation (Pastrana et al., 1985; Blanco et al., 1986, 1988). Initiation activation requires the formation of the protein p6 complex with Φ29 DNA terminal fragments (Serrano et al., 1989) and it was suggested to undergo through transient unwinding of DNA at the p6 specific binding sites that would favor interaction of the TP/DNAP complex with the template strand (Serrano et al., 1993b). In line with this hypothesis, Φ29 origins with partially unpaired ends showed increased utilization (up to 30-fold respect to wild type origins) (Gella et al., 2014). Initiation of these modified origins was still stimulated by p6, although to a lesser extent (around 1.5-fold) than the wild type origin (2.8-fold).

As mentioned above, p6 is also important for the control of transcription, either by itself or together with the viral transcriptional regulator p4 (Camacho and Salas, 2000, 2001). Thus, protein p6 switches off very early transcription from promoter C2, as shown by *in vivo* and *in vitro* studies, impairing the RNA polymerase complex access to the nucleoprotein complex at the promoter region (Serrano et al., 1989; Camacho and Salas, 2001). Moreover, formation of p6 nucleoprotein complex promotes p4-mediated repression of promoters A2b and A2c and activation of the A3 promoter (Calles et al., 2002).

In the context of the infected cell, p6 is highly abundant, which would favor oligomerization and formation of p6 nucleocomplex (Abril et al., 1997), which might be even more favored under the crowded intracellular environment (Alcorlo et al., 2009). This high density is in agreement with a histone-like function that would complex with the entire genome (Serrano et al., 1994; Holguera et al., 2012), analogously to cellular histones. At early infection stages, p6 localizes mainly in a peripheral helix-like configuration (Holguera et al., 2012), whereas the viral genome and the replication machinery associates with the host nucleoid (Muñoz-Espín et al., 2010). Since protein p6 is essential to initiate Φ29 DNA replication, it was suggested that a small amount of protein p6 (undetectable by immunofluorescence) would be recruited early at the bacterial nucleoid, establishing the appropriate conditions at the phage DNA ends to achieve the first rounds of replication. Then, and as viral DNA replication progresses, p6 is recruited to the bacterial nucleoid and, by topological recognition of the Φ29 DNA, avoids its sequestration by the higher volume of the bacterial DNA. Under this scenario, Φ29 p6 constitutes a histone-like protein specific for the viral genome, whose temporal and spatial subcellular localization is determined by its essential roles in genome replication and transcription (Holguera et al., 2012).

## Role of host DNA-binding proteins in Φ29 DNA replication

Bacteriophages have developed different strategies to inactivate or take advantage of cellular enzymes in their own benefit (Roucourt and Lavigne, 2009). During Φ29 infection several *B. subtilis* DNA-binding proteins have been shown to play a role in the development of the infective cycle.

### DNA gyrase

Chromosomal DNA topology is controlled by various host-encoded topoisomerases, such as DNA gyrase (topoisomerase II) (Drlica, 1992). Despite containing a TP covalently linked to the 5′ ends and therefore not being covalently closed, Φ29 DNA is topologically constrained *in vivo* (González-Huici et al., 2004c). In this sense, it has been shown that the gyrase inhibitor novobiocin but not nalidixic acid, which also inhibits DNA gyrase but does not have topological effects on DNA, increases the binding of protein p6 to the viral genome *in vivo*. In addition, both novobiocin and nalidixic acid impair viral DNA replication *in vivo*, suggesting that *B. subtilis* gyrase is involved in viral DNA replication (González-Huici et al., 2004c). A topologically constrained DNA should be allowed to rotate freely during the replication process, explaining the necessity of the bacterial DNA gyrase (Muñoz-Espín et al., 2012). Moreover, Φ29 genome possesses two convergently oriented transcription units encompassing genes 7–16 and 17–16.5. Hence, without the action of DNA gyrase, a highly positive supercoiled region would be generated between the two convergently oriented transcription units, preventing the advance of the RNA polymerase, and/or inducing DNA polymerase template switching when encountering this blockage. In fact, during Φ29 infection subgenomic viral DNA molecules ranging from 1 to 8 Kb are accumulated, originated mainly from the right end of the genome and that these kind of molecules do not accumulate when *B. subtilis* cells are infected with the transcription deficient mutant Φ29 *sus4*(56), which does not express protein p4 (Murthy et al., 1998).

The topological constraint of bacteriophage Φ29 genome could be achieved by binding of the parental TPs either directly to the nucleoid or to the bacterial membrane through the interaction with other viral proteins such as p1 or p16.7 (see above) (Bravo et al., 2000; Serna-Rico et al., 2003; Muñoz-Espín et al., 2010).

### Uracil-DNA glycosylase

A potential threat to genome integrity is the presence of uracil residues in DNA. Uracil is eliminated from DNA genomes by the base excision repair pathway (BER), which is initiated with the enzymatic activity of a uracil-DNA glycosylase (UDG). These enzymes (Family 1) selectively remove uracil bases from both single- and double-stranded DNA, cleaving the N-glycosidic bond between the base and the deoxyribose, leaving therefore an abasic site (Savva et al., 1995). This abasic site is then repaired through the sequential action of an apurinic/apyrimidinic endonuclease, DNA polymerase and DNA ligase. Most eukaryotic and prokaryotic cells encode a UDG to maintain the integrity of DNA genomes. However, there are some cases in which the presence of uracil in DNA could be desirable. For instance, *B. subtilis* phage PBS1 (and its clear-plaque isotype PBS2) genome contains uracil instead of thymine and, consequently, encodes a UDG inhibitor (called Ugi) to assure an efficient viral genome replication (Katz et al., 1976; Cone et al., 1980; Savva and Pearl, 1995). Additionally, phage T5 infection induces an inhibitor of *E. coli* UDG that has not yet been identified (Warner et al., 1980). Interestingly, despite having a non-uracil containing genome, phage Φ29 encodes a UDG inhibitor, a small acidic protein of 56 amino acids called p56 (Serrano-Heras et al., 2006). Protein p56 is expressed early after infection and interacts with *B. subtilis* UDG, inhibiting its activity (Serrano-Heras et al., 2006). *In vitro* experiments showed that protein p56 blocks the DNA-binding ability of UDG, and structural data suggest that it does it by mimicking the structure of DNA (Serrano-Heras et al., 2007; Asensio et al., 2011; Baños-Sanz et al., 2013; Cole et al., 2013). As mentioned above, the mechanism of Φ29 DNA replication involves the generation of replicative intermediates that contain large stretches of ssDNA (Harding and Ito, 1980; Inciarte et al., 1980; see Figure 1). If uracil residues were present in these stretches of ssDNA by either the misincorporation of deoxyuridine monophosphate (dUMP) during the replication process or by the spontaneous deamination of cytosine in DNA, the elimination of these lesions by the BER pathway would give rise to the loss of terminal viral DNA regions. In fact, it has been shown that Φ29 DNA polymerase can incorporate dUMP during DNA synthesis with a catalytic efficiency of only 2-fold lower than dTMP, and perform the extension of base-paired uracil residues to give full-length DNA *in vitro* (Serrano-Heras et al., 2008). Hence, by encoding an UDG inhibitor, Φ29 prevents the elimination of uracil residues that could be present in the ssDNA portions of the genome replicative intermediates and that would compromise viral genome integrity (Serrano-Heras et al., 2006; Muñoz-Espín et al., 2012).

It is worth mentioning that Φ29-related phages PZA, B103, Nf, and GA-1 encode homologs of p56. The product of GA-1 gene *56* was purified and shown to inhibit UDG activity in extracts of both *B. subtilis* and *B. pumilus*, which is the natural host of GA-1 (Pérez-Lago et al., 2011).

The elucidation of the function of several Φ29 proteins yet to be characterized and the improvement of *in vivo* techniques for both protein-protein and protein-DNA interactions detection will lead to a better understanding of the virus-host interactome in the future.

## Author contributions

All authors listed have made substantial, direct and intellectual contribution to the work, and approved it for publication.

## Funding

This work has been supported by grants from the Spanish Ministry of Economy and Competitiveness (BFU2014-52656-P to MS) and (BFU2014-53791-P to MV), ComFuturo Grant from Fundación General CSIC (to MR) and by an Institutional grant from Fundación Ramón Areces to the Centro de Biología Molecular “Severo Ochoa.”

### Conflict of interest statement

The authors declare that the research was conducted in the absence of any commercial or financial relationships that could be construed as a potential conflict of interest.
